# Ectopic pure mediastinal parathyroid adenoma: A case report

**DOI:** 10.1016/j.ijscr.2021.106598

**Published:** 2021-11-12

**Authors:** Hanan M. Hemead, Ahmed Abdelaziz Abdellatif, Mostafa A. Abdel Rahman

**Affiliations:** Alexandria Faculty of Medicine, Champollion Street, Al Mesallah Sharq, Al Attarin, Alexandria Governorate, Egypt

**Keywords:** Thoracoscopic surgery, Video assisted, Parathyroid neoplasms, Mediastinum, Case report

## Abstract

**Introduction and importance:**

The ectopic parathyroid adenoma is an important cause of refractory and recurrent hyperparathyroidism. The mediastinal location of ectopic parathyroid tissue is reported in up to 20% of cases of ectopic parathyroid adenomas. In around 2% of cases where cervical approach is unfeasible, the mediastinal route imposes a surgical challenge.

**Case presentation:**

We describe a case of a twenty-five-year-old male patient with manifestations of hyperparathyroidism. Computed tomography of the chest showed an anterior mediastinal mass. Nuclear scintigraphy detected a functioning parathyroid tissue in the mediastinum. The mass was excised en block with the surrounding adipose tissue using a three-port video-assisted thoracoscopic surgery. The patient showed a full symptomatic and laboratory recovery.

**Clinical discussion:**

Mediastinal parathyroid glands comprise a unique surgical entity with diagnostic and management difficulties.

**Conclusion:**

Mediastinal parathyroid gland is a rare yet important cause of refractory hypercalcemia. The current localization tools improve the thoracoscopic management of MPAs. VATS can provide access and exposure to ectopic parathyroid adenoma with low morbidity.

## Highlights

• Ectopic mediastinal parathyroid glands are rare yet challenging cause of refractory or recurrent hypercalcemia.
• Most mediastinal parathyroids are approachable through a neck incision.
• Larger, deeper or totally mediastinal glands require thoracic access.
• Historically, in challenging cases, sternotomy or thoracotomy is performed with associated high morbidity in some cohorts.
• The video-assisted thoracoscopic surgery (VATS) allows surgeons to retrieve these glands guided with localization tools.

## 1 Introduction

Primary hyperparathyroidism is characterized by excessive secretion of parathyroid hormone due to parathyroid adenoma, hyperplasia or rarely a parathyroid cancer. Asymptomatic presentation is not uncommon [1]. Surgical treatment remains the benchmark therapy. Effective surgery depends mainly on identification and adequate removal of parathyroid glands which was historically performed through bilateral neck exploration due to lack of localization technique. Refractory or recurrent primary hyperparathyroidism is attributed to inadequate resection or the development of second adenoma or cancer. The presence of an ectopic parathyroid adenoma; however, constitutes another important cause particularly with the competence of surgeons to perform parathyroidectomy with a success rate exceeding 90% [2].

Ectopic parathyroid adenomas result from migratory misbehavior during embryogenesis. The inferior parathyroid glands originate from the 3rd brachial arch which also gives rise to the thymus gland and accompany it during its descent. Therefore, deviation from the normal pathway of descent results in their ectopia anywhere in the mediastinum up to the pericardium. The superior parathyroid glands arise from the 4th branchial arch and follow the thyroid gland during its caudal migration. Mediastinal parathyroid adenomas (MPAs) are found in up to 20% of patients with ectopic parathyroid glands. In around 2%, the cervical retrieval is unfeasible as the adenoma ≥6 cm or deeply seated in the mediastinum [3]. In these circumstances, the mediastinal route becomes inevitable. Evolution of high-resolution computed tomography (CT), magnetic resonance imaging (MRI) and nuclear scintigraphy facilitates minimally-invasive approach through accurate localization. The video-assisted thoracoscopic surgery (VATS) offers an excellent modality combining the merits of a minimally invasive approach and magnification of the surgical field [4]. This case report has been reported in line with the SCARE Criteria [5].

## 2 Case presentation and methods

**Fig. 1:**
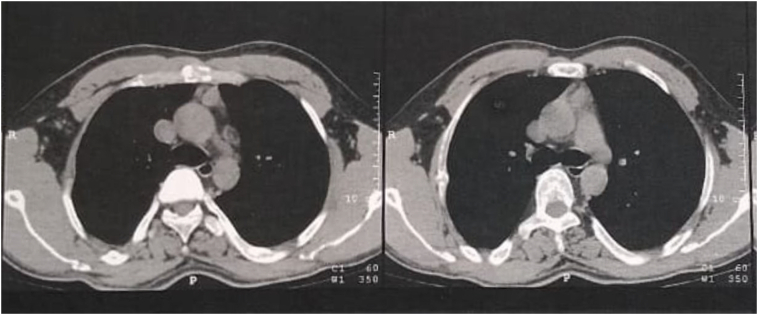
CT chest showing anterior mediastinal mass.

**Fig. 2:**
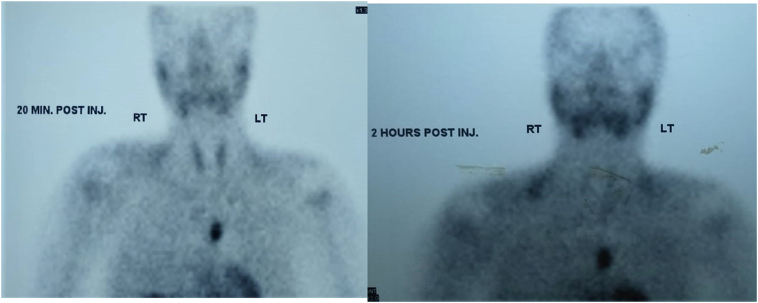
^99m^Tc-MIBI showing increased uptake in the mediastinum after 20 min and the delayed image showing no evidence of washout.

A 25-year-old male patient presented with a four-month history of abdominal pain, renal stones and recent recurrent pathological fractures rendering him wheelchair bound. Past medical history was unremarkable, and no previous intervention was performed. The patient denied any allergies and family history was unremarkable. Physical examination was free. Investigations revealed hypercalcemia and hyperparathyroidism with hypophosphatemia. The neck ultrasound was unremarkable. Non-contrast CT of neck and chest detected an anterior mediastinal mass (Fig. 1). The ^19m^Tc-methoxyisobutylisonitrile scintigraphy (^99m^Tc-MIBI) showed increased uptake of the tracer in the mediastinum (Fig. 2). The patient consented to have his information disclosed in this case report.

**Fig. 3:**
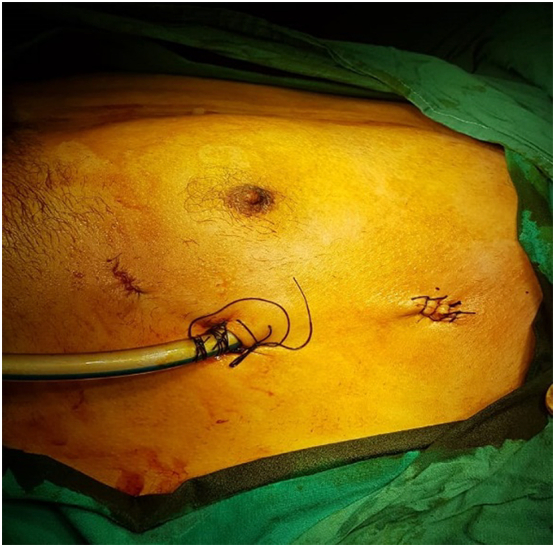
Incisions made for introduction of camera and working instruments.

**Fig. 4:**
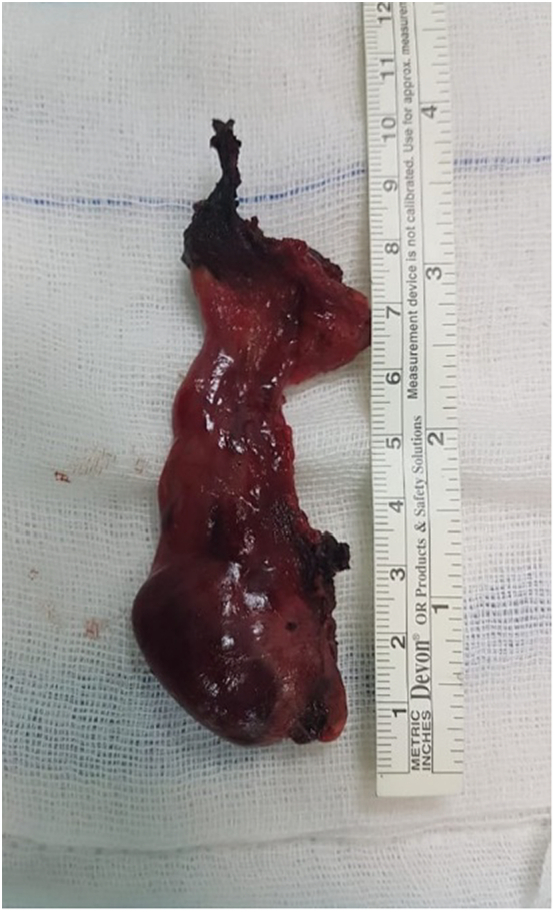
The excised mass.

The patient was positioned in the semi-supine position with the arms abducted and the left side elevated. Endocrinological review was performed prior to surgery to optimize electrolyte levels. The procedure was performed by a consultant thoracic surgeon in our institute which is a tertiary care hospital. Three-port thoracoscopy was performed on the left side using a thirty-degree 10 mm thoracoscope (Karl Storz, Tuttlinger, Germany). Working ports were placed in the 3rd and 6th intercostal spaces at the anterior axillary line and the camera port in the 4th intercostal space at the mid-axillary line (Fig. 3). Careful inspection of the left phrenic nerve and the thymic area harboring the mass was performed. Dissection was done using monopolar energy and the mass excised en-block (Fig. 4). A chest drain was inserted. The procedure was uneventful with no postoperative complications.

## 3 Results

Pathological examination confirmed the presence of parathyroid adenoma. Serological and symptomatic recovery was achieved, and the patient was discharged in the third postoperative day. Follow up in 1st, 3rd and 6th months postoperatively was performed, patient was seen in outpatient clinics for physical examination and serological tests which revealed normal calcium and parathyroid hormone. When asked about his symptoms, he was satisfied with the resolution of symptoms.

## 4 Discussion

MPAs comprise a unique surgical entity with diagnostic and management difficulties. MPAs are either originally has developed in the neck and descended to mediastinum or most commonly originally developed in the mediastinum. In the 1–2% of cases were mediastinal exploration is needed, median sternotomy or anterior thoracotomy were the main approaches used and were associated with a high failure rate approaching 40% and sternal-related complications in 20% of cases. Less aggressive interventions such as; transcervical approach with sternal retraction, subxiphoid laparoscopy and angiographic ablation were attempted. The available localization techniques in that era were computed tomography which yielded high false negative results and parathyroid arteriography which carries neurological risks. Additionally, the angiographic ablation was associated with recurrence, early failure and hypoparathyroidism [6]. The current localization tools improve the thoracoscopic management of MPAs. VATS can provide access and exposure to ectopic parathyroid adenoma with low morbidity. Though we did not apply that in our case, the intraoperative assay of parathormone is a critical tool to assess the success of the procedure, the adequacy of resection and the need of neck exploration.

## 5 Conclusion

When MPAs are detected preoperatively with localization tools, VATS is a safe and effective approach obviating the need of sternotomy.

## Funding

This research did not receive any specific grant from funding agencies in the public, commercial, or not-for-profit sectors.

## Provenance and peer review

Not commissioned, externally peer-reviewed

## Ethical approval

Institutional Board Review (IBR) was obtained.

## Consent

Written informed consent was obtained from the patient for publication of this case report and accompanying images. A copy of the written consent is available for review by the Editor-in-Chief of this journal on request.

## Author contribution

1st and 2nd authors: conceptualizing, data collection, validation, investigation, data curation, writing final draft, 3rd author: data validation, investigation, curation, writing and revising the final version, supervision.

## Research registration

N/A.

## Guarantor

Hanan M. Hemead, Ahmed Abdelaziz.

## Declaration of competing interest

None.
